# Real-time measurements of spontaneous breathers and rogue wave events in optical fibre modulation instability

**DOI:** 10.1038/ncomms13675

**Published:** 2016-12-19

**Authors:** Mikko Närhi, Benjamin Wetzel, Cyril Billet, Shanti Toenger, Thibaut Sylvestre, Jean-Marc Merolla, Roberto Morandotti, Frederic Dias, Goëry Genty, John M. Dudley

**Affiliations:** 1 Tampere University of Technology, Department of Physics, Optics Laboratory, FI-33101 Tampere, Finland; 2 Institut National de la Recherche Scientifique (INRS), Centre EMT, Université du Québec, Varennes, Québec, Canada J3X 1S2; 3 Department of Physics and Astronomy, School of Mathematical and Physical Sciences, University of Sussex, Sussex House, Falmer, Brighton BN1 9RH, UK; 4 Institut FEMTO-ST, CNRS Université de Bourgogne Franche-Comté UMR 6174, 25030 Besançon, France; 5 Institute of Fundamental and Frontier Sciences, University of Electronic Science and Technology of China, Chengdu 610054, China; 6 National Research University of Information Technologies, Mechanics and Optics, St. Petersburg, Russia; 7 School of Mathematics and Statistics, University College Dublin, Belfield, Dublin 4, Ireland

## Abstract

Modulation instability is a fundamental process of nonlinear science, leading to the unstable breakup of a constant amplitude solution of a physical system. There has been particular interest in studying modulation instability in the cubic nonlinear Schrödinger equation, a generic model for a host of nonlinear systems including superfluids, fibre optics, plasmas and Bose–Einstein condensates. Modulation instability is also a significant area of study in the context of understanding the emergence of high amplitude events that satisfy rogue wave statistical criteria. Here, exploiting advances in ultrafast optical metrology, we perform real-time measurements in an optical fibre system of the unstable breakup of a continuous wave field, simultaneously characterizing emergent modulation instability breather pulses and their associated statistics. Our results allow quantitative comparison between experiment, modelling and theory, and are expected to open new perspectives on studies of instability dynamics in physics.

## Abstract (web summary)

Low amplitude noise on an otherwise constant-intensity wave can grow exponentially and induce nonlinear dynamical behaviour. Here, the authors present time-domain measurements of a phenomenon arising from such modulation instability: the emergence of highly localised breathers in an optical fibre.

Dynamical instabilities are seen in many areas of physics, and their study has major applications in physics, chemistry, biology and the social sciences^1^. An especially important class of instability is the ‘modulation instability' (MI) which describes how low-amplitude noise on an initial wave of constant intensity can grow exponentially and induce a wide range of nonlinear dynamical behaviour. Although first seen in deep-water wave propagation described by the cubic nonlinear Schrödinger equation (NLSE) (where it was referred to as the Benjamin–Feir instability)^2^, MI has attracted particularly widespread interest in optics and has been observed in a variety of nonlinear systems. The first observation of MI in optics was in optical fibre propagation described by the cubic NLSE^3^,^4^, but other classes of related instabilities have since been reported in laser resonators and optical cavities^5^,^6^,^7^,^8^,^9^,^10^, spatio-temporal dynamics^11^,^12^, pattern formation^13^,^14^,^15^,^16^,^17^ and waveguides^18^.

Despite much research into more general manifestations of MI in optics, the cubic NLSE remains the canonical system of interest that illustrates the essential characteristics of the phenomenon^19^,^20^,^21^. In addition, because the cubic NLSE describes pulse envelope propagation both in optical fibre and on the surface of deep water, there has been particular attention paid to the analogy between the instability growth dynamics in fibre optics and the formation of extreme rogue waves on the ocean^22^,^23^,^24^. Indeed, although nonlinear noise amplification has been considered as a possible ocean rogue wave generation mechanism for some time^25^,^26^, this possibility attracted renewed attention following experiments in optics where real-time measurements showed long-tailed statistics in the spectral intensity of an optical fibre supercontinuum^27^. These experiments motivated significant wider interest in the statistical properties of random processes in optics, and subsequent studies have investigated long-tailed statistics in a variety of other optical systems^28^, including those with only linear elements^29^. It is important to stress, however, that the link between such ‘optical rogue waves' and ocean waves remains an open question^30^.

A significant feature of MI in the cubic NLSE arises from the fact that the low-amplitude noise on the initial conditions can be treated as a perturbation, allowing linear stability analysis to be used to derive the proper conditions for the excitation and growth of the instability. Moreover, beyond the initial phase of exponential instability growth, the MI dynamics lead to the generation of highly localized pulses that can be described in terms of analytic families of soliton on finite background or ‘breather' solutions^31^. In fact, although these analytic solutions were known since the 1980s^32^, it is only very recently that advances in optical measurement technologies have actually permitted these solutions to be observed in experiments^33^,^34^,^35^,^36^,^37^. However, these previous experiments were carried out in the regime of ‘induced MI,' where a low-amplitude narrowband stable modulation on a continuous wave was used to stimulate the instability dynamics from a coherent seed^4^. Although highly significant from the perspective of confirming the analytic theory of breathers^31^, these experiments do not, however, model the scenario of the ‘spontaneous' MI where breather-like rogue waves emerge from low-amplitude broadband noise. Yet, many numerical studies have shown that the analytic breather solutions of the NLSE can in fact also describe the localized structures emerging from spontaneous MI arising from a noisy continuous wave as initial condition^24^,^38^,^39^,^40^.

A number of experiments studying spontaneous MI in cubic-NLSE systems have been carried out, but studies of irregular water waves have focussed primarily on statistical measures, and have not considered wave envelope properties in detail^26^,^41^. In optical fibre, the experiments have generally been limited to using only time-averaged autocorrelation to characterize the unstable field envelope^3^,^42^, although real-time measurements of the spectral fluctuations have been possible using dispersive Fourier transformation^21^,^43^. Important recent experiments have reported the use of optical sampling^44^ and a time-lens system^45^ to study the evolution that arises from the propagation of an irregular high-contrast pulse train seed in an optical fibre^46^,^47^. From a dynamical perspective, the absence of a significant continuous wave component in the initial conditions leads to a very different propagation regime from MI. The higher energy associated with these initial conditions leads to random background-free fundamental and higher-order soliton evolution^40^,^48^ whose intensity profile can reach locally extreme values, and where the central peak may be fitted with a Peregrine soliton (PS) structure^46^,^47^. Although different from the regime of MI dynamics arising from low-amplitude broadband noise that we study here, this work shows how real-time optical techniques can be advantageously used to characterize the full field probability density^44^ and intensity profiles^45^ of background-free soliton time series.

In this paper, we report time-domain measurements of highly localized breathers generated from the spontaneous MI of a continuous wave (CW) field in a cubic NLSE optical fibre system. Using an integrated time-lens magnifier system^49^,^50^, we capture in real time an extended series of transient high intensity breather pulses emerging from noise. The large data set allows quantitative comparison between measured statistics and those obtained from Monte Carlo NLSE simulations, and intensity envelope measurements confirm that the properties of the spontaneously generated breather profiles seen in experiment are in excellent agreement with analytic predictions. From a physical viewpoint, this agreement allows us to confirm experimentally that spontaneous MI can be interpreted in terms of breather solutions of the cubic NLSE, and by comparing peak-to-background ratios obtained from experiments with theory, we are also able to identify the most extreme events as corresponding to their collisions. Aside from representing a major advance in the experimental study of ultrafast MI, our results open up new possibilities for the study of other classes of nonlinear dynamical processes in optics.

## Results

### Numerical simulations of modulation instability dynamics

We first review the theory of MI and present numerical results showing the expected time-domain breather dynamics. The starting point is the cubic NLSE written in the notation of nonlinear fibre optics:


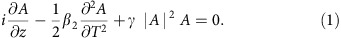


Here *A*(*z*,*T*) is the pulse envelope in a co-moving frame (at the envelope group velocity) and *γ* (W^−1^ m^−1^) and *β*_2_ (s^2^ m^−1^) are the fibre nonlinear coefficient and group velocity dispersion respectively. The units of *A*(*z*,*T*) are W^1/2^, with 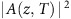 yielding instantaneous power. Note that the NLSE is derived assuming a perturbative expansion of the material nonlinear response which is fully justified at the power levels used in our experiment^4^.

**Figure 1:**
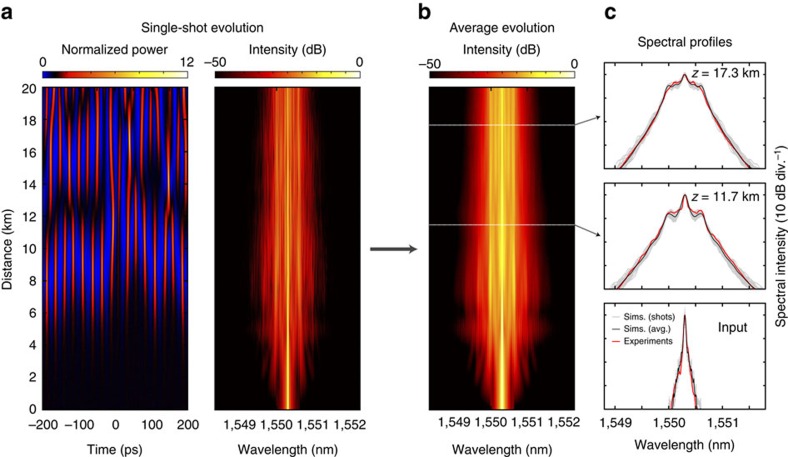
Temporal and spectral evolution of modulation instability. (**a**) Single-shot simulations showing temporal and spectral evolution of spontaneous MI. (**b**) Evolution of the average spectrum calculated from an ensemble of 50 simulations. (**c**) compares the simulated average spectrum (black) with that measured experimentally (red) at the fibre input and after 11.7 and 17.3 km propagation as indicated. For each case in **c**, the superimposed grey curves also plot 50 individual realizations from the simulations to illustrate the degree of spectral fluctuation. For the chaotic temporal pulse train, we plot instantaneous pulse power normalized to background 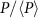, whilst spectral plots are normalized to the input spectral intensity at 1550.3 nm.

Linear stability analysis assuming a low-amplitude modulation can be used to show that a constant-intensity wave of power *P*_0_ is unstable when *β*_2_<0, and the instability exhibits maximum gain at modulation frequency 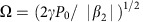 (ref. 4). Figure 1 shows results from numerical simulations of the MI process with fibre parameters corresponding to our experiments at input wavelength of 1550.3 nm: 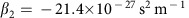 and 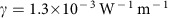. We simulate propagation in km-lengths of fibre of a noisy continuous wave input field with *P*_0_=0.7 W. The input noise model includes the effect of amplified spontaneous emission (ASE) and phase modulation in the initial conditions, but no forcing noise source during propagation (for example, spontaneous Raman scattering) was included. With narrowband initial conditions and our parameters, the intensity contrast on the input continuous wave is <5% ensuring that we are in a low noise regime where the expected dynamics of cubic-NLSE MI can be clearly observed^4^,^40^. Note also that simulations were performed with higher-order dispersive and nonlinear terms, but these effects were found to be negligible (see ‘Methods' section).

Figure 1a plots typical simulation results showing field temporal and spectral evolution for a single numerical realization. The temporal evolution plot clearly reveals the emergence of distinct breather pulses from the injected continuous wave background as a result of MI, and we stress that it is precisely the intensity profiles of these (randomly evolving) structures that have never been measured before. We also note that although our simulations include loss to facilitate quantitative comparison with experiments, the expected NLSE breather growth and decay dynamics are very clearly observed. The associated spectral evolution shows the initial stage of MI sideband generation at frequency Ω/2*π*=46.4 GHz (≈0.37 nm) for *P*_0_=0.7 W.

Performing multiple simulations for different random noise seeds (see ‘Methods' section) allows us to plot the evolution of the average spectrum as shown in Fig. 1b. Figure 1c compares the computed average spectra from simulations (black) with experimentally measured spectra (red) at three propagation distances. The superimposed grey curves also plot 50 individual realizations from the simulations to illustrate the degree of spectral fluctuation observed. Note that for comparison with experiments, simulation results are convolved with the resolution response of the optical spectrum analyser (OSA) used in our setup (see ‘Methods' section). There is excellent correspondence between the experimental and simulated average spectra, and we highlight in particular the agreement in the spectral wings over more than 30 dB. In this context, it is important to note that the particular ‘triangular' nature of these wings (when viewed on a semi-logarithmic scale) is a characteristic feature of the emergence of temporal breathers, and is an important confirmation that our experiments are being performed in the regime of MI in the cubic-NLSE^20^,^35^.

### Real-time measurement of emerging localized structures

**Figure 2:**
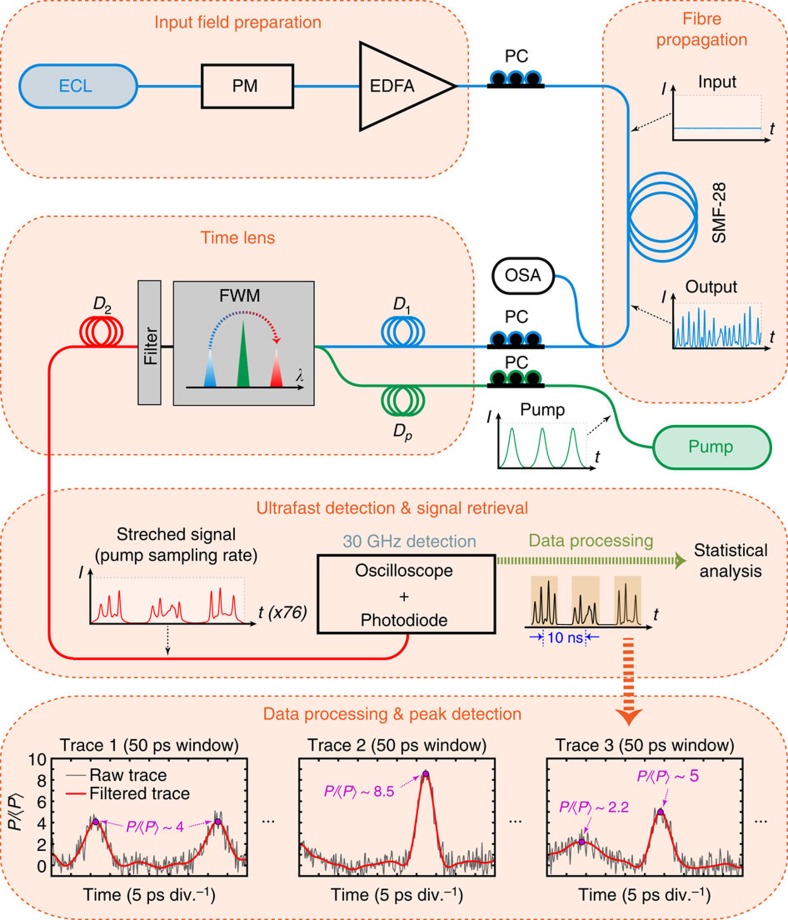
Experimental setup. An external cavity laser (ECL) at 1550.3 nm is phase modulated (PM) and amplified in an erbium-doped fibre amplifier (EDFA) before injection in SMF-28 single-mode fibre. The input noisy continuous wave breaks up into a random breather train which is measured using an optical spectrum analyser (OSA) and a time-lens magnifier (PicoLuz UTM-1500). The time lens uses two dispersive propagation steps (*D*_1_ and *D*_2_), one on each side of an element that applies a quadratic temporal phase. In our experiments, the quadratic phase was applied from four wave mixing (FWM) with a linearly chirped pump pulse co-propagating in a silicon waveguide. The chirped pump pulses of duration ∼90 ps are generated from a femtosecond pulse fibre laser at 100 MHz subsequently stretched through propagation in a fibre of dispersion *D*_*p*_. Optimal fidelity in the time lens requires polarization control (PC) for the pump-signal FWM in the silicon waveguide. The wavelength-converted idler after FWM (with applied quadratic phase) is filtered before further propagation in segment *D*_2_. The overall temporal magnification is 76.4 such that the stretched replica of the input at the output of *D*_2_ can be resolved using a 30 GHz bandwidth detection system (photodiode and ultrafast oscilloscope). The output signal takes the form of a sequence of noisy breather peaks separated by 10 ns. Note that the figure plots results over a timespan of 50 ps, but peak detection used only a 25 ps region of the measurement window. We also show three representative signals obtained from experiment (at 17.3 km propagation) plotting both unfiltered raw data (black traces), and data after filtering (see ‘Methods' section) to remove instrumental noise (red curves).

To characterize these breathers experimentally, we used the setup in Fig. 2. A continuous wave 1550.3 nm external cavity laser (ECL) was phase modulated (to suppress Brillouin scattering^33^,^51^) and amplified to *P*_0_=0.7 W before injection in standard single-mode fibre in which the MI develops (see ‘Methods' section). The figure also illustrates schematically the fact that the instability develops from an injected continuous wave as initial condition. At this power, simulations indicate that the unstable pulses have expected durations in the range 2–12 ps which we measured using the time-lens system described below^50^.

When applied to a continuous wave field, the time-lens measurements yield distinct segments of the noisy pulse structure at a repetition rate of 100 MHz. After correcting for magnification, the physical (that is, demagnified) width of the measurement window was ∼50 ps. For peak detection and analysis, we consider a smaller region of 25 ps at the centre of the measurement window, from which it is straightforward to extract intensity profiles of individual breather pulses. The digital oscilloscope used to record the traces introduces a low level of noise spanning the complete bandwidth of our detection system (out to 40 GHz) with a constant level and, to improve signal fidelity, we apply a frequency-domain numerical filter which allows us to more clearly identify the maxima and determine the temporal widths of the signal peaks (see ‘Methods' section). Three subplots in Fig. 2 show both unfiltered raw data (black traces) and filtered data (red curves) for representative signals obtained from our experiments, illustrating how the filtering procedure is effective at allowing us to identify peak maxima. Note that during the peak detection process over the central 25 ps region of the measurement window, only peaks that showed distinct maxima and subsidiary minima were included in the statistical analysis. See ‘Methods' section for further details of the time-lens system.

**Figure 3:**
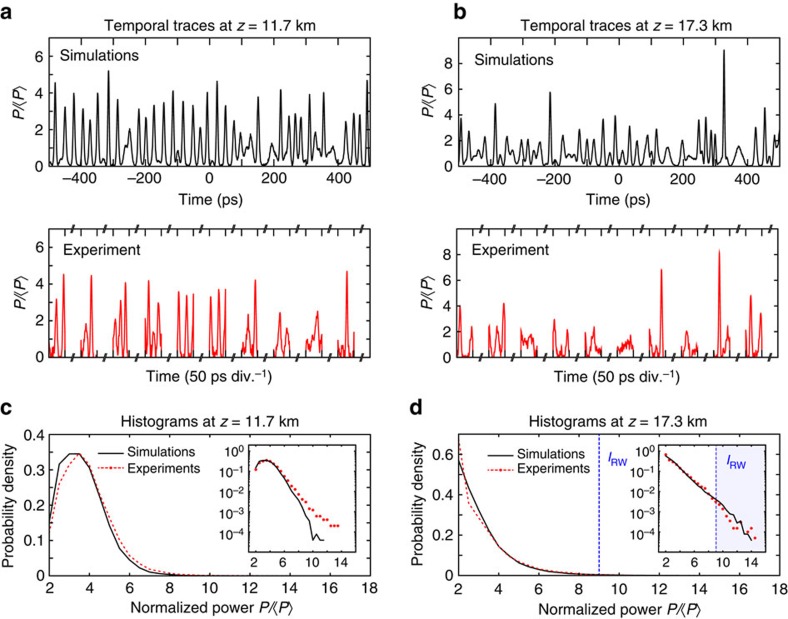
Time lens results and histograms. Experimental results at (**a**) 11.7 km and (**b**) 17.3 km showing intensity profiles from simulations (top, black) and experiments (bottom, red) as indicated. Plotted intensities *P* are normalized with respect to the average output background power 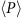 and plotted against time after rescaling to account for the effect of magnification. The experimental results are shown for sequential measurement windows (note the broken axis between measurements). (**c**,**d**) Histograms of the peak intensities (shown as normalized probability density) from the chaotic pulse trains at 11.7 and 17.3 km respectively, comparing experiment (red) and simulation (black). The insets plots the histograms on semi-logarithmic axes. The calculated rogue wave intensity threshold (*I*_RW_) is shown as a dashed blue line.

Figure 3 compares experiment and simulation characterizing MI at two different fibre lengths of (a) 11.7 km and (b) 17.3 km. For each case we show a time series of intensity fluctuations obtained from simulations (top, black), and a section of a corresponding intensity time series from experiments (bottom, red). Although the experimental measurements are sampled at the repetition rate of the time-lens pump (indicated by the broken time axis), we see nonetheless good visual agreement in the general characteristics of the observed intensity fluctuations. These results complement those shown in the traces of Fig. 2 in illustrating the general features of a noisy MI field with random breathers. Note that while single isolated breathers can be observed in some instances, in many cases the pulse structure is more complex, but this is expected in the regime of spontaneous MI where breathers generated from noise will overlap and merge^39^.

Note that these results plot 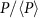, the measured signal normalized with respect to the measured average background CW power at the fibre output, which facilitates comparison with expected analytic solutions, and which provides an important measure that can be used to infer details of the underlying dynamics^24^,^38^,^39^. In particular, in the regime of MI in the cubic NLSE, the peak-to-background ratio 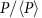 can be used to distinguish elementary breathers from collision events between breathers, because it is well-known that the highest possible peak-to-background of an elementary (single) breather is 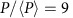 corresponding to the PS of the NLSE^52^. The only physical mechanism that can possibly yield a ratio 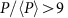 is therefore a collision between elementary breathers^31^. This criterion to distinguish elementary breather solutions from collision events has already been used in several works^38^,^53^.

### Statistical analysis

The statistics of these results are characterized by computing histograms of the peak intensities of the pulses seen in the time series^30^. Figure 3 plots such histograms (equivalent probability density) comparing experiments (red) and simulations (black) for (c) 11.7 km and (d) 17.3 km propagation respectively. The inset plots use a semi-logarithmic axis. The histograms show clear qualitative differences with a distinct peak in the distribution for the 11.7 km case, and a near-exponential distribution at 17.3 km. This is consistent with the fact that 11.7 km is close to the first compression point of the MI evolution where more uniformity in pulse height might be expected. We also note that peaks with normalized powers exceeding the single breather PS limit of 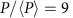 (ref. 31) are observed in both cases. Experiments and simulations at both fibre lengths match well up to this limit, and there is also very good agreement in Fig. 3d above the PS limit for 17.3 km propagation. The discrepancy in modelling the statistics above the PS limit in Fig. 3c is attributed to uncertainties in modelling the input noise; the effect of the initial conditions on the evolution is expected to be less important for longer distances when the dynamics are dominated by turbulence^54^.

From these statistics, it is straightforward to calculate the rogue intensity threshold *I*_RW_ which defines the intensity above which events can be classified as rogue waves in the accepted statistical sense (see ‘Methods' section). At the longest propagation distance of 17.3 km, when we are in a regime of turbulent dynamics, the blue dashed line in Fig. 3d shows the calculated value of *I*_RW_=9.2 from the experimental data, very close to the value of *I*_RW_=9.1 calculated from the simulation results. We also note that the fraction of events above *I*_RW_ can be readily calculated from experiment at ∼0.3%, a small fraction consistent with previous predictions for NLSE systems^24^,^39^.

Note that with measurements made at a fixed fibre distance, the MI breather profiles are not necessarily characterized at their point of maximum intensity, but rather at various points along their longitudinal evolution. However, although the associated statistics will differ from those obtained considering peaks in a two-dimensional NLSE field^39^, we can nonetheless draw an important conclusion about the physical nature of events that exceed the rogue wave threshold. In particular, since the calculated *I*_RW_ is very close to the limiting value of 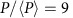 above which peaks in the MI field must necessarily arise from breather collisions (see above)^31^, this implies that the events classified as rogue waves in our data are associated with breather collisions. This is an important aspect of our study: the link between rogue wave events and breather collisions in the cubic NLSE has previously been made through numerical simulations^24^,^39^,^40^, and our results allow us to confirm this experimentally.

### Comparison with analytical breather theory

**Figure 4:**
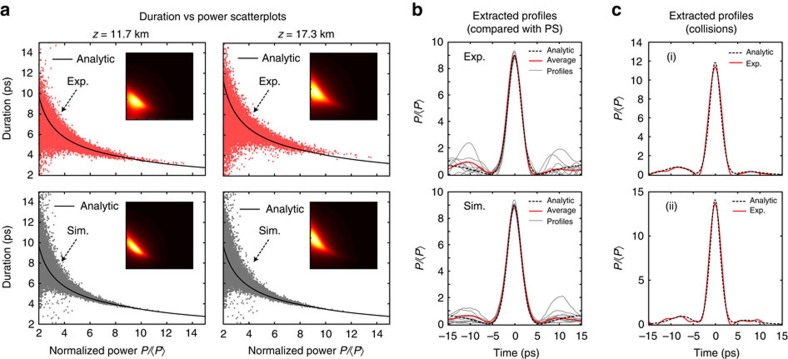
Temporal properties of breathers. (**a**) Scatter plots of pulse duration against normalized power from experimental intensity profiles (top, red points) and simulation profiles (bottom, grey points) for 11.7 and 17.3 km propagation respectively, compared with theory (black line) in each case. The insets show the scatter plot converted to a false-color density map. (**b**) Results extracting 10 intensity profiles at 11.7 km with 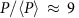 (grey lines) from experiment (top) and simulation (bottom). The average intensity profiles (red lines) are compared with the analytic PS (black dashed lines), showing excellent agreement over the central peak. (**c**) Two peaks of higher intensity (grey lines) with (i) 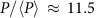 and (ii) 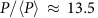. The dashed line in each case shows a higher-order breather fit.

The time-lens measurements also permit quantitative comparison of the properties of the breather intensity profiles from experiments and those expected from theory. Firstly, for 30,000 distinct pulses in the time series, Fig. 4a plots the pulse duration (full-width at half-maximum(FWHM)) against the corresponding normalized peak power comparing experiment (top, red points) and simulation (bottom, grey points) at two propagation distances as shown. As expected from a noise-driven process, there is significant scatter, but the results from experiment and simulation agree well, and both cluster strongly around the theoretical prediction relating duration to power based on the analytic properties of NLSE elementary breathers and their collisions (see ‘Methods' section). (Because of the large number of points in the figure, the insets also show the scatter plot converted to a false-color density map.) These experimental results are important in confirming previous suggestions that the analytic predictions of the NLSE provide a natural basis with which to interpret the temporal structures in MI, even when stimulated from noise^39^.

We can also compare the intensity profiles of the measured breather pulses with simulation and theory, and these results are presented in Fig. 4b,c. Firstly, from experimental and simulated data, we select intensity profiles with 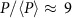 and a well-defined central peak with near 100% contrast (that is, zero intensity on either side of the central maxima). Figure 4b shows 10 such intensity profiles from experiment (top) and simulation (bottom). Although both cases show large fluctuations in the pulse structure in the wings (which is to be expected given that they emerge from noise), the central peaks overlap very closely. In addition, in the region of the central peak, the mean intensity profiles from experiment and simulation (red lines) are in excellent agreement with the analytic PS (black dashed lines) solution calculated for our parameters (see ‘Methods' section). Note that when we measure at a fixed distance, the selection of peaks with 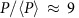 will include not only ideal PSs at their maxima, but also some breather collision events with maximal intensity ≈9. But we can attribute the excellent agreement with the PS profile due to its universal role in nonlinear dynamics that has been shown to also apply to a range of other propagation scenarios^46^,^47^.

For structures with intensities 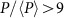 arising from breather collisions, there is no simple analytic description. Nonetheless, it is possible to construct the theoretical profile of a breather collision using the Darboux transformation^31^. Two examples of experimentally measured collision profiles are shown in the grey lines in Fig. 4c for a normalized power of 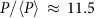 (top) and 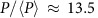 (bottom). Note that these are single-shot measurements and not averages over an ensemble. In both cases we compare these results with theory by using the Darboux transformation to analytically construct a second-order breather solution (see ‘Methods' section) and the construction is in excellent agreement with experiment. It is important to bear in mind that, in a noisy MI field, all the Fourier modes underneath the MI gain bandwidth are simultaneously excited such that the resulting evolution corresponds to the nonlinear superposition of the structures associated with these Fourier modes. This implies that the fit of a particular analytical breather (or collision thereof) can generally be performed only locally to a limited interval of the temporal field profile. To identify all the structures that are present in the random MI field generated in an integrable cubic NLSE system, a different approach consists in using the inverse scattering transform to compute the eigenvalue spectrum associated with a particular evolving field, allowing discrimination between particular structures that can emerge along propagation^26^,^40^,^55^.

## Discussion

There are several important conclusions to be drawn from this work. Firstly, our results show direct measurements of the ultrafast intensity profiles of optical breather structures emerging from noise-driven MI, and our measurements confirm analytical predictions made decades ago but never previously quantified in any physical system. We have been able to extract profiles of pulses satisfying criteria as statistical rogue waves, and our results have allowed us to associate these with collisions between elementary NLSE breathers. Our combined time domain, spectral and statistical characterization provides a comprehensive view of the underlying dynamics of MI and nonlinear breather formation that develop from the exponential amplification of broadband random noise in a cubic NLSE system.

Furthermore, these results complement the recent experimental study of transient intense structures that can emerge from a broadband input field corresponding to a random high-contrast pulse train^45^, and whose evolution is naturally described in terms of random (background-free) higher-order soliton dynamics^40^. We anticipate that a detailed comparison between the long-term evolution and turbulence properties of these two regimes could provide additional insights into nonlinear propagation dynamics in optical fibres with noisy initial conditions.

Finally, we also note that our results highlight the intrinsic advantages of using advanced pulse metrology techniques in optics to continuously revisit and retest different scenarios in nonlinear optics. Our successful use of the time-lens approach to measure MI opens up possibilities in characterizing other optical systems displaying instabilities such as lasers with nonlinearity and feedback^56^, or ultrafast lasers in which dissipative soliton evolution takes place^57^. We expect that time-lens measurements will become a standard technique in allowing robust tests of theory and numerical modelling in many other dynamical systems in optics. We anticipate that the next important step towards a complete characterization and understanding of the dynamics of integrable nonlinear optical systems will be to access the complex amplitude associated with a given propagating field, which would then allow to unambiguously identify signatures of particular analytical structures using computational techniques such as, for example, the inverse scattering transform^26^,^40^,^55^.

## Methods

### Numerical simulations

Although the NLSE equation (1) describes the qualitative MI dynamics very well for our experimental regime, for completeness in simulations, we used a generalized form of the NLSE including higher-order dispersion, inelastic Raman scattering, self-steepening and loss^58^. Simulation parameters were: 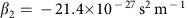, 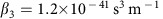, 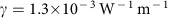, linear loss of 0.18 dB km^−1^ and 0.3 dB connector loss. Note that we checked using the generalized NLSE that there were negligible differences in simulations performed with and without the higher-order nonlinear terms, confirming the interpretation of our results in terms of cubic NLSE dynamics. In fact, experimental evidence for the negligible influence of higher-order dispersion and higher-order nonlinear effects is also seen in the symmetrical spectral broadening and linear spectral wings (on a semi-logarithmic scale) in Fig. 1. We also note that some effect of loss can be seen in simulations leading to a slight decrease in mean spectral bandwidth with propagation, but this has no effect on the qualitative growth and decay breather dynamics in the time domain.

Initial conditions used a continuous wave field of power *P*_0_=0.7 W modified by phase modulation and with ASE noise to model the contribution from the erbium-doped fibre amplifier. The phase modulation is applied to suppress Brillouin scattering when using continuous wave excitation, and we used a standard technique^51^ with a ∼G bit s^−1^ pseudo-random bit sequence of ±*π* phase swings which was included in the simulations. The ASE noise was included through a spectral background of −50 dB relative to the pump with random spectral phase. The spectral background level corresponded to that measured experimentally. The statistics of the MI spectral and temporal properties were obtained by Monte Carlo simulations to generate an ensemble of simulations using different random number seeds. The numerical ensemble yielded typically ∼50,000 distinct intensity peaks from which a large number of distinct random peaks and a corresponding intensity histogram could be readily obtained for comparison with experiment.

### Experimental setup

An ECL (Agilent 81949A) at 1550.3 nm was first phase-modulated using an electro-optic phase modulator driven by a pseudo-random bit sequence pattern generator (Tektronix AWG7122C) before amplification in an EDFA (Keopsys C40-PB) and coupling into single-mode fibre SMF-28. We performed initial experiments over a wide range of input powers (0.3–1.2 W) but the evolution and breather characteristics were found to be qualitatively similar, and the results we present here at *P*_0_=0.7 W are typical. An optical circulator was used to monitor Brillouin backscattered light which was always <5% of the input power. Spectral measurements used an OSA (Anritsu OSA MS9710B) with 0.07 nm resolution.

The time lens used was a commercial Picoluz UTM-1500 system, similar to that described in ref. 50. Total accumulated dispersion for the input and output propagation steps was: *D*_1_=4.16 ps nm^−1^ (−5.32 × 10^−24^ s^2^) and *D*_2_=318 ps nm^−1^ (−406 × 10^−24^ s^2^) respectively, with magnification 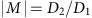. The temporal quadratic phase (to reproduce the effect of a thin lens) was imposed through four wave mixing from a pump pulse (100 MHz Menlo C-Fiber Sync and P100-EDFA) with linear chirp accumulated from propagation in a pre-chirping fibre segment *D*_*p*_. The imaging condition for magnification is 2/*D*_*p*_=1/*D*_1_+1/*D*_2_, so that the dispersion for the pump is around twice that of the signal input step. The time-lens output was detected with a 38 GHz photoreceiver (New Focus 1474-A) connected to a 30 GHz channel of a real-time oscilloscope (LeCroy 845 Zi-A 80 GS/s). Combining the electronic detection bandwidth with the optical parameters of the time-lens yields an overall intrinsic demagnified temporal resolution of <300 fs (ref. 50).

The measurement window in magnified time was ≈5 ns, and raw data traces are shown in Fig. 2 for a region of magnified time of 3.9 ns, corresponding to 50 ps in demagnified time. (Note that all results in Figs 3 and 4 are plotted against demagnified time.) For peak detection with improved signal-to-noise and to avoid any possibility of overlap between sequential records, we considered only the central 25 ps region (in demagnified time) of the measurement window. These measurements were then normalized to the mean background intensity to yield a peak-to-background ratio 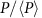 for comparison with simulations. The mean background for normalization was obtained by measuring the time-lens output with a continuous wave signal at the same average power but with no SMF-28. This measurement was also verified by taking the average over a large number of the random MI pulse samples in the MI regime.

The digital oscilloscope used to record the traces introduces a low level of background noise (∼mV) which can be seen on the unfiltered data (black traces) shown in Fig. 2. This broadband noise consists of fast fluctuations at the oscilloscope sampling interval of 12.5 ps extending up to a frequency of 2/12.5 ps=40 GHz in a single sideband spectrum (limited by the sampling rate of the oscilloscope). This sampling noise was filtered in the Fourier domain with a sixth-order super-Gaussian low-pass filter with a 8 GHz FWHM bandwidth. After accounting for the effects of magnification, this yields an effective bandwidth of 610 GHz for resolving physical structures on the MI field. The appropriate bandwidth of the RF filter applied to reject unwanted noise was determined from the noise of the detection system both in the absence of any signal and when a narrowband CW field was directly measured. We further checked that the filtering operation did not distort pulse measurements by characterizing coherently seeded breather structures. The seeded breathers were generated from a modulated signal^33^,^36^,^37^ with various modulation periods leading to compressed structures with durations in the range 2–12 ps similar to those observed in the spontaneous MI experiments, and which could be compared with ideal analytical breather solutions.

Inspection of the results in Fig. 2 reveals visually how the filtering procedure retains the overall peak structure very well when applied to experimental breather pulses. We also tested the effect of the filtering more quantitatively using simulated data of a spontaneous MI breather train to which sampling noise (as in experiment) was added numerically. We found that the intensity and temporal duration of the filtered peaks was within 5% of the original noise-free for breathers of duration greater than 3 ps duration, which in fact represents over 99% of the extracted peaks expected from simulations (see simulation results in Fig. 4). This testing of the filtering using numerical data also confirmed that artifacts such as ringing or peak asymmetry were not introduced by the procedure.

### Fitting experimental data

The results in Fig. 4 plot temporal duration (FWHM) against normalized power 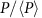 from an ensemble of ∼30,000 distinct pulse profiles from experiments, and ∼50,000 distinct pulse profiles from simulation. The theoretical curve shown in the figure is calculated from the known properties of the elementary Akhmediev breather for 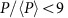 (refs 31, 39) and for the second-order solution for 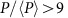 corresponding to the in-phase collision between two elementary breathers constructed using the Darboux transformation technique^31^. Note that the theoretical curves plot duration against normalized power at the point of maximum temporal localization, but it is clear from Fig. 4 that the scattered points extracted from the random MI field cluster around the theoretical curve. The intensity *I*_RW_ indicated on the histograms describes a threshold criterion used to distinguish the small number of high intensity rogue wave events from the general population of intensity peaks. This is calculated from analysis of the statistics of the intensity peaks to determine the ‘significant intensity' *I*_1/3_ which is the mean intensity of the highest third of events. The rogue wave intensity threshold is then defined as *I*_RW_=2*I*_1/3_, a criterion that is generally accepted as it can be calculated for any underlying probability distribution^30^.

The detailed analytic breather profiles that are compared with experiments in Fig. 4b,c are based on well-known theory of NLSE breathers^31^. Figure 4b plots the analytic result for the PS^52^. Plotting the normalized intensity 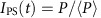 against the physical time *t*, the analytic PS formula is 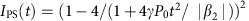 where the parameters are as given above. Note there are no free parameters in the calculation of the PS profile which is compared with the experimental data. Also note that the experimental and simulation data in Fig. 4b show 10 randomly extracted peaks with maxima within 5% of the PS limit of 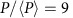. The collision event in Fig. 4c was fitted using a three-parameter fit describing the analytic superposition of two elementary Akhmediev breathers^31^. Specifically, the temporal profile corresponding to the collision of two breathers can be fully described by the temporal periods of each breather train and their relative spatial (longitudinal) phase, and it is these parameters that were fitted using a least-squares method^31^. The fit in Fig. 4c (i) used two breather trains of periods 22.9 and 37.0 ps with longitudinal separation of 98.6 m and the fit in Fig. 4c (ii) used two breather trains of periods 27.1 and 30.5 ps with longitudinal separation of 19.7 m. We see remarkable agreement between the experimentally measured temporal profile of the extreme events and that of the second-order breather fit. We also emphasize that equally good fits are obtained for other events in the tail of the histograms of Fig. 3. These results provide clear evidence that: (i) the analytic AB solutions of the NLSE provide an appropriate basis for describing the nature of localized structures that emerge from a chaotic MI field and (ii) the structures with highest intensity arise from the collision of elementary breathers. We stress that there is a fundamental difference between elementary and high-order breathers. An elementary breather corresponds to the exponential growth and decay of a weakly modulated continuous background with a single modulation frequency. On the other hand, a collision of breathers arises when two different elementary breather structures are simultaneously excited and their relative phase is such that the distances at which they reach maximum intensity closely coincide. A collision can thus also be seen as the nonlinear superposition of elementary breather structures.

### Data availability

The data that support the findings of this study are available from the corresponding author upon request.

## Additional information

**How to cite this article:** Närhi, M. *et al*. Real-time measurements of spontaneous breathers and rogue wave events in optical fibre modulation instability. *Nat. Commun.* **7,** 13675 doi: 10.1038/ncomms13675 (2016).

**Publisher's note:** Springer Nature remains neutral with regard to jurisdictional claims in published maps and institutional affiliations.
